# Neudesin as a unique secreted protein with multi-functional roles in neural functions, energy metabolism, and tumorigenesis

**DOI:** 10.3389/fmolb.2015.00024

**Published:** 2015-05-19

**Authors:** Hiroya Ohta, Ikuo Kimura, Morichika Konishi, Nobuyuki Itoh

**Affiliations:** ^1^Department of Microbial Chemistry, Kobe Pharmaceutical UniversityKobe, Japan; ^2^Department of Applied Biological Science, Graduate School of Agriculture, Tokyo University of Agriculture and TechnologyTokyo, Japan; ^3^Department of Genetic Biochemistry, Kyoto University Graduate School of Pharmaceutical SciencesKyoto, Japan

**Keywords:** anxiety, membrane-associated progesterone receptor, metabolism, neudesin, neuron-derived neurotrophic factor, obesity, tumorigenesis

## Abstract

Neudesin was originally identified as a secreted protein with neurotrophic activity, and, thereafter, was also termed neuron-derived neurotrophic factor (NENF) or the candidate oncogene GIG47. Neudesin with a conserved cytochrome 5-like heme/steroid-binding domain activates intracellular signaling pathways possibly through the activation of G protein-coupled receptors. In the brain, hypothalamic *Neudesin* decreases food intake. *Neudesin* knockout (KO) mice also exhibit anxiety-like behavior, indicating its roles in the hippocampal anxiety circuitry. *Neudesin* is also expressed in various peripheral tissues. *Neudesin* KO mice are strongly resistant to high-fat diet (HFD)-induced obesity due to elevated systemic sympathetic activity, heat production, and adipocytic lipolysis. *Neudesin*, which is over-expressed or induced by DNA hypomethylation in multiple human cancers, also stimulates tumorigenesis. These findings indicate that Neudesin plays roles in neural functions, energy metabolism, and tumorigenesis and is expected to be a novel target for obesity and anti-cancer treatments.

## Introduction

**Figure 1:**
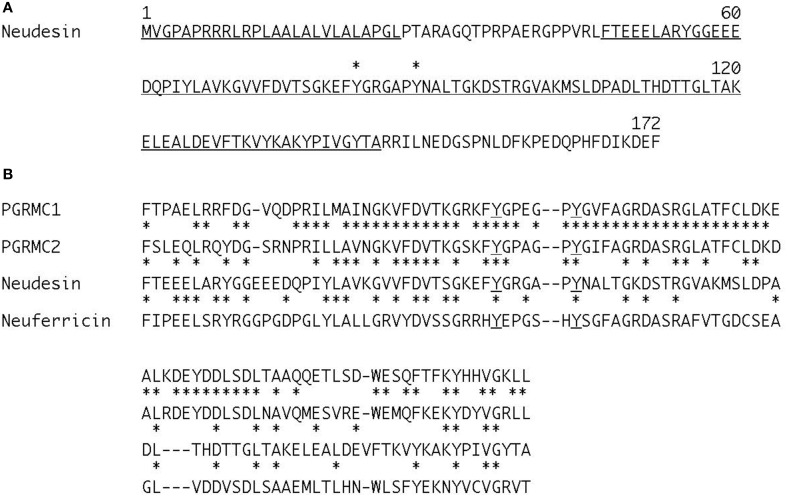
**Amino acid sequence of human Neudesin (A) and comparison of heme/steroid-binding domains in the human MAPR family (B). (A)** Underlines indicate a cleavable secreted signal sequence and conserved heme/steroid-binding domain. Asterisks indicate the two conserved tyrosine residues, 82 and 88, which are essential for heme-binding. The numbers refer to amino acid positions. **(B)** Dashes indicate gaps introduced to align sequences. Asterisks indicate identical amino acid residues in sequences. Underlines indicate the two conserved tyrosine residues essential for heme-binding in the MAPR family.

Neudesin was originally identified as a secreted protein with neurotrophic activity from mouse embryos (Kimura et al., 2005). Thereafter, it was also termed NENF and GIG47 (Han et al., 2012; Byerly et al., 2013). Human Neudesin is a secreted protein of 172 amino acids with a conserved cytochrome 5-like heme/steroid-binding domain of ~100 amino acids (Figure 1A) (Kimura et al., 2005, 2008). Neudesin is a member of the membrane-associated progesterone receptor (MAPR) protein family comprising three additional members with characteristic cytochrome 5-like heme/steroid-binding domains: progesterone receptor-membrane component (PGRMC1), PGRMC2, and Neuferricin (Figure 1B) (Ohta and Itoh, 2012; Kimura et al., 2013).

PGRMC1 was originally identified as a putative receptor for progesterone (Meyer et al., 1996). However, PGRMC1 binds heme, not progesterone (Cahill, 2007). PGRMC1 and PGRMC2 are mainly located in the endoplasmic reticulum (Gerdes et al., 1998; Chen et al., 2010). The expression of *PGRMC1* is up-regulated in cancer cells. PGRMC1 promotes cell survival and damage resistance in cancer cells and also plays roles in lipid, drug, and hormone metabolism in the liver and neuroprotection in the brain. *PGRMC2* is also expressed in breast adenocarcinoma (Rohe et al., 2009; Intlekofer and Petersen, 2011). In contrast, Neuferricin is a secreted protein that promotes neurogenesis in neural precursor cells and suppresses cell survival in Neuro2a cells (Kimura et al., 2010). Neuferricin also enhances the survival of ectoside-exposed cells, cytochrome P450 activities, and the growth and survival of HeLa cells (Xie et al., 2011; Bruce and Rybak, 2014).

*PGRMC1* and *PGRMC2* have two conserved introns, indicating that they were generated from a common ancestral gene. In contrast, *Neudesin* has non-conserved introns. Thus, Neudesin is not evolutionarily related to the other members of the MAPR family (Ohta and Itoh, 2012). The roles and action mechanism of Neudesin are also distinct from those of the other members. Neudesin plays roles as a multi-functional secreted protein in neural functions, energy metabolism, and tumorigenesis. In this review, we focused on multi-functional Neudesin with recent findings.

## Structure

The amino acid sequence of Neudesin is highly conserved in vertebrates (Ohta and Itoh, 2012). A nuclear magnetic resonance analysis indicated that Neudesin, which is also termed GIG47, has an α-helice/β-strand structure with a β 1-α1-β 2-β 3-α2-β 4-α3-α4-β 5-β 6 topology. The heme/steroid-binding domain is located in the α2-β 4-α3 topology. A homology modeling calculation with the known tertiary structure of 1TOG, a hypothetical protein of unknown function with a cytochrome b5-like fold, indicated the tertiary structure of Neudesin. A potential heme/steroid-binding hydrophobic pocket is visible between the α2 and α3 helices. Tyrosine residues 82 and 88 in this pocket are essential for heme-binding (Han et al., 2012).

## Activity and action mechanism of neudesin

*Neudesin* is preferentially expressed in the brain and spinal cord at embryonic stages in mice, but is widely expressed in various tissues including the brain, adipose tissue, heart, lung, and kidney at postnatal stages. In the brain, *Neudesin* is preferentially expressed in neurons. Neudesin exhibits significant neurotrophic activity in primary cultured neurons, but not mitogenic activity in primary cultured astrocytes, indicating that it is a neurotrophic factor. The conserved cytochrome 5-like heme/steroid-binding domain of Neudesin has been shown to bind heme and is required for its activity (Kimura et al., 2005, 2008). Neudesin activates the mitogen-activated protein kinase (MAPK) and phosphatidylinositol-3 kinase (PI3K) pathways. The phosphorylation of extracellular signal-regulated kinase (ERK)1/2 by Neudesin is inhibited by the pertussis toxin (PTX), an inhibitor of the Gi/Go-protein, indicating that its activity is mediated via activation of the MAP and PI3K pathways, which are potentially coupled with the Gi/Go-protein-coupled signaling pathway (Kimura et al., 2005) (Table 1).

**Table 1:** **Activity and roles of Neudesin in neural functions, energy metabolism, and tumorigenesis**.

**Neudesin gain-of-function**	**Loss-of-function**	**Action site**	**References**
**NEURAL FUNCTIONS**			
Neurotrophic activity		Cultured neuronal cells	Kimura et al., 2005
Differentiation activity		Cultured neural precursor cells	
Cell proliferation activity		Cultured neural precursor cells	Kimura et al., 2006
	Inhibition of cell proliferation/survival	Cultured Neuro2a cells	Kimura et al., 2008
Decreased food intake		Mice	Byerly et al., 2013
	Anxious-like behavior	Mice	Ohta et al., 2015
**ENERGY METABOLISM**			
Inhibition of adipogenesis		Cultured 3T3-L1 cells	Kimura et al., 2009
	Promotion of adipogenesis	Cultured 3T3-L1 cells	Kimura et al., 2009
	Resistance to HFD-induced obesity/metabolic dysfunction	Mice	Ohta et al., 2015
	Increased sympathetic activity	Mice	Ohta et al., 2015
	Increased energy expenditure	Mice	Ohta et al., 2015
	Increased heat production/fatty acid oxidation in BAT	Mice	Ohta et al., 2015
	Increased lipolysis in WAT	Mice	Ohta et al., 2015
**TUMORIGENESIS**			
Invasiveness activity		Cultured MCF cells	Han et al., 2012
Tumorigenicity		Cultured MCF cells	Han et al., 2012
	Inhibition of cell growth	Cultured cancer cells	Stefanska et al., 2014
	Inhibition of invasiveness	Cultured cancer cells	Stefanska et al., 2014

*Neudesin* is also expressed in neural precursor cells before the appearance of neurons in mice, indicating its potential roles in neural development (Kimura et al., 2006). Neudesin significantly promotes neuronal differentiation that is mediated though activation of the protein kinase A (PKA) and PI3K pathways in cultured neural precursor cells. It also transiently promotes neural cell proliferation early in the developmental process. This proliferation is mediated through the MAPK and PKA pathways. The expression profile and activity of Neudesin indicate that it plays unique roles in neural cell proliferation and neuronal differentiation (Kimura et al., 2006). The phosphorylation of ERK, serine-threonine protein kinase AKT, and cAMP response element binding protein (CREB) is also promoted by Neudesin in neural precursor cells. However, its action is not inhibited by PTX. Neudesin increases cAMP levels in neural precursor cells, indicating that its activity is potentially exerted via the Gs protein-coupled signaling pathway and that the mechanism of action of neudesin in these cells is distinct from that in the neurons (Kimura et al., 2006). *Neudesin* is also abundantly expressed in cultured mouse neuroblastoma Neuro2a cells. Cell survival and proliferation are significantly decreased in Neuro2a cells by the siRNA-mediated knockdown of *Neudesin* (Kimura et al., 2008). Neudesin activates the MAPK and PI3K signaling pathways in cultured adipocytes and cancer cells (Kimura et al., 2009; Han et al., 2012) (Table 1).

Hemin significantly promotes the activity of Neudesin in primary cultured cells, indicating that the heme-binding domain is essential for its activity (Cahill, 2007). As Neudesin is a secreted protein, it is expected to exert its activity by binding to and activating its specific cell surface receptor. Since the activity of Neudesin is exerted via the G protein-coupled signaling pathway, its receptor is expected to be a G protein-coupled receptor (GPCR). Although currently unknown, the identification of Neudesin receptors will provide an important insight into the action mechanism of Neudesin.

## Roles of neudesin in neuronal functions

Neudesin exhibits neurotropic activity in cultured neuronal cells, therefore, it is assumed to play *in vivo* roles in neuronal functions. Some secreted proteins in the brain including brain-derived neurotrophic factor (BDNF), ciliary neurotrophic factor, and neuropeptide Y can regulate energy uptake and expenditure (Fargali et al., 2012). Neudesin was also identified through large-scale screening aimed at identifying novel secreted hypothalamic proteins that regulate food intake (Byerly et al., 2013). *Neudesin* is strongly expressed in hypothalamic nuclei that regulate food intake. BDNF in the hypothalamus is an important regulator of appetite. The expression of *Neudesin* in the hypothalamus is decreased by the administration of BDNF into the lateral cerebral ventricle. The administration of Neudesin into the cerebral ventricle resulted in decreases in food intake and body weight and increases in the expression of *pro-opiomelanocortin* and *melanocortin 4 receptor* in the hypothalamus. However, the effects of Neudesin on food intake is abrogated in obese mice fed a high-fat diet (HFD), indicating the diet-dependent modulation of Neudesin functions. These findings indicate that hypothalamic Neudesin is a potential central modulator of food intake via a regulatory circuit that may involve BDNF and melanocortin signaling (Byerly et al., 2013) (Table 1).

*Neudesin* knockout (KO) mice appear to be normal and fertile (Novais et al., 2013; Ohta et al., 2015). An extensive behavioral characterization (motor, emotional, and cognitive dimensions) of *Neudesin* KO mice revealed anxiety-like behavior. In association with the anxious phenotype, dopaminergic input was reduced and dendritic arborization was impoverished in dentate gyrus granule neurons in the ventral hippocampus. Shorter dendrites were also observed in the bed nucleus of the stria terminalis. These findings suggest the roles of Neudesin in maintaining the hippocampal anxiety circuitry (Novais et al., 2013) (Table 1).

## Roles of neudesin in energy metabolism

White adipose tissue (WAT) is crucially involved in energy metabolism. Obesity, which is characterized by the excessive development of WAT, is a risk factor for several metabolic diseases, including type II diabetes, hypertension, and atherosclerosis. The development of WAT involves adipogenesis and an increase in the number of cells (Hausman et al., 2001). *Neudesin* is also abundantly expressed in the WAT of mice. It has been shown to significantly suppress adipogenesis in cultured 3T3-L1 preadipocytes. The knockdown of *Neudesin* by RNA interference markedly promoted adipogenesis by suppressing activation of the MAPK pathway activation in 3T3-L1 cells. These findings suggest that Neudesin may be a negative regulator in the early stage of adipogenesis (Kimura et al., 2009) (Table 1).

*Neudesin* KO mice are strongly resistant to HFD-induced obesity and metabolic dysfunction. However, food intake is essentially unaffected in *Neudesin* KO mice fed HFD (Ohta et al., 2015). Thus, resistance to HFD-induced obesity is independent of food intake. As discussed above, the administration of Neudesin decreased food intake (Byerly et al., 2013). This discrepancy may be explained by differences in physiological analyses using *Neudesin* KO mice and pharmacological analyses by the administration of recombinant Neudesin.

Adipose tissue consists of two distinct types: white and brown. WAT stores excess energy as triglycerides. In contrast, brown adipose tissue (BAT) dissipates energy as heat, thereby counteracting obesity (Frontini and Cinti, 2010; Cristancho and Lazar, 2011). The sympathetic nervous system (SNS) plays crucial roles in maintaining energy homeostasis. The activated SNS stimulates lipolysis in WAT and enhances heat production in BAT by activating adrenergic signaling. Thus, the SNS is essential for regulating adipose function and the development of obesity (Tentolouris et al., 2006). Sympathetic activity was found to be significantly increased in *Neudesin* KO mice fed HFD, resulting in increased energy expenditure and heat production as well as fatty acid oxidation in BAT and enhanced lipolysis in WAT (Ohta et al., 2015) (Table 1). These findings indicate that Neudesin is a negative regulator of energy expenditure and could be an attractive target for the development of anti-obesity drugs.

## Roles of neudesin in tumorigenesis

*Neudesin* has also been identified as GIG47 using a differential display technique to discover genes critical for breast tumorigenesis (Han et al., 2012). *Neudesin* is over-expressed in multiple human cancers including carcinomas of the breast, uterine cervix, malignant lymphoma, colon, lung, and skin as well as in leukemia and breast cancer cell line MCF-7. The ectopic expression of *Neudesin* in MCF7 cells has been shown to promote invasiveness *in vitro* and increase tumorigenicity *in vivo*. The mechanism underlying tumorigenesis may be mediated by activation of the MAPK and PI3K pathways. These findings indicate that Neudesin is involved in tumorigenesis and may be a novel target for the treatment of cancers (Table 1).

Common hypomethylated genes in many cancers are candidates for novel broad-spectrum anti-cancer and anti-metastatic agents. Whole-genome mapping has identified many activated gene promoters by DNA hypomethylation in hepatocellular carcinoma (HCC) clinical samples. *Neudesin* is also hypomethylated and induced in HCC (Stefanska et al., 2014). The ineffectiveness of *Neudesin* mediated by RNA interference in different types of cancers effectively and specifically inhibited their cell growth and invasive capacities. This ineffectiveness was also found to reduce their growth as explants in mice and interfere with the AKT, WNT, and MAPK signaling pathways. These findings indicate that *Neudesin* is induced by hypomethylation in many cancers and is a candidate target for anti-cancer therapeutics in multiple cancer cell types (Table 1).

## Conclusions

Neudesin, which is a member of the MAPR family, is a unique secreted protein with a conserved cytochrome 5-like heme/steroid-binding domain and plays multi-functional roles in neural functions, energy metabolism, and tumorigenesis. In the brain, Neudesin may be a neurotrophic factor in food intake in the hypothalamus and in maintaining the hippocampal anxiety circuitry. *Neudesin* KO mice are strongly resistant to HFD-induced obesity, indicating that Neudesin is a negative regulator of energy expenditure in peripheral tissues. *Neudesin* is also expressed in multiple human cancers and stimulates tumorigenesis. Further studies on Neudesin will provide useful clues for the development of treatments for metabolic diseases and cancers.

### Conflict of interest statement

The authors declare that the research was conducted in the absence of any commercial or financial relationships that could be construed as a potential conflict of interest.
